# Standardized Video Interviews Do Not Correlate to United States Medical Licensing Examination Step 1 and Step 2 Scores

**DOI:** 10.5811/westjem.2018.11.39730

**Published:** 2018-12-12

**Authors:** Daniel J. Egan, Abbas Husain, Michael C. Bond, William Caputo, Lukasz Cygan, Jeff Van Dermark, Jan M. Shoenberger, Ida Li, William Krauss, Jonathan Bronner, Melissa White, Arlene S. Chung, Kaushal H. Shah, Todd Taylor, Matthew Silver, Brahim Ardolic, Moshe Weizberg

**Affiliations:** * Columbia University Vagelos College of Physicians and Surgeons, Department of Emergency Medicine, New York, New York; † Staten Island University Hospital – Northwell, Department of Emergency Medicine, Staten Island, New York; ‡ University of Maryland School of Medicine, Department of Emergency Medicine, Baltimore, Maryland; § University of Texas Southwestern Medical Center, Department of Emergency Medicine, Dallas, Texas; ¶ University of Southern California, Keck School of Medicine, Department of Emergency Medicine, Los Angeles, California; || Kaiser Permanente San Diego Medical Center, Department of Emergency Medicine, San Diego, California; # University of Kentucky, Department of Emergency Medicine, Lexington, Kentucky; ** Emory University, Department of Emergency Medicine, Atlanta, Georgia; †† Maimonides Medical Center, Department of Emergency Medicine, Brooklyn, New York; ¶¶ Icahn School of Medicine at Mount Sinai Hospital, Department of Emergency Medicine, New York, New York

## Abstract

**Introduction:**

In 2017, the Standardized Video Interview (SVI) was required for applicants to emergency medicine (EM). The SVI contains six questions highlighting professionalism and interpersonal communication skills. The responses were scored (6–30). As it is a new metric, no information is available on correlation between SVI scores and other application data. This study was to determine if a correlation exists between applicants’ United States Medical Licensing Examination (USMLE) and SVI scores. We hypothesized that numeric USMLE Step 1 and Step 2 Clinical Knowledge (CK) scores would not correlate with the SVI score, but that performance on the Step 2 Clinical Skills (CS) portion may correlate with the SVI since both test communication skills.

**Methods:**

Nine EM residency sites participated in the study with data exported from an Electronic Residency Application Service (ERAS®) report. All applicants with both SVI and USMLE scores were included. We studied the correlation between SVI scores and USMLE scores. Predetermined subgroup analysis was performed based on applicants’ USMLE Step 1 and Step 2 CK scores as follows: (≥ 200, 201–220, 221–240, 241–260, >260). We used linear regression, the Kruskal-Wallis test and Mann-Whitney U test for statistical analyses.

**Results:**

1,325 applicants had both Step 1 and SVI scores available, with no correlation between the overall scores (p=0.58) and no correlation between the scores across all Step 1 score ranges, (p=0.29). Both Step 2 CK and SVI scores were available for 1,275 applicants, with no correlation between the overall scores (p=0.56) and no correlation across all ranges, (p=0.10). The USMLE Step 2 CS and SVI scores were available for 1,000 applicants. Four applicants failed the CS test without any correlation to the SVI score (p=0.08).

**Conclusion:**

We found no correlation between the scores on any portion of the USMLE and the SVI; therefore, the SVI provides new information to application screeners.

## INTRODUCTION

Residency program directors (PD) screen large volumes of applications each recruitment season. A significant portion of each application is subjective, leaving ambiguity in data interpretation. Additionally, the medical student performance evaluation (MSPE) includes selected quotations from clinical clerkships and may or may not make a summative comparison of students to their peers. Emergency medicine (EM) has attempted to standardize recommendation letters and clerkship-grading transparency through the Standardized Letter of Evaluation (SLOE).^1^ Without standardization, letters of recommendation showed grade inflation, lack of meaningful comparison between applicants, and the inability to use them as discriminatory tools for success in residency.^2^–^4^ Inconsistencies in grades and evaluations by gender have also been demonstrated in other specialties.^5^ Even with standardized letters in several specialties, the use of the full spectrum of global assessments has not been found consistently nor has the accurate prediction of an applicant’s position on the rank list.^1^,^6^,^7^

The only fully objective data on the residency application are the licensing examinations (United States Medical Licensing Examination [USMLE] and the Comprehensive Osteopathic Medical Licensing Examination [COMLEX]). These exams allow for direct applicant comparison as opposed to grades, which vary between schools. USMLE Step 2 Clinical Skill (CS) and COMLEX Step 2 Performance Evaluation (PE) require medical students to perform a history and physical examination on standardized patients. Step 2 CS “uses standardized patients to test medical students on their ability to gather information from patients, perform physical exams and communicate their findings to patients and colleagues.”^8^ These exams also incorporate communication skills to patients and colleagues into the final pass/fail grade. In its rationale for the Step 2 CS portion, the USMLE reports that poor communication and interpersonal skills are a reason for complaints against physicians. The scores on this portion of the examination also predict the success of these skills in first-year residents.^9^

During the 2017–2018 application season, the Association of American Medical Colleges (AAMC) instituted the Standardized Video Interview (SVI) as part of the EM residency application process. Using six questions, the SVI sought to provide objective information related to interpersonal and communication skills, and knowledge of professional behaviors.^10^ Applicants answered each question for up to three minutes, and a trained rater scored each video on a scale of 1–5, yielding a summative score of 6–30. Trained raters used anchors based on behavioral examples defining the proficiency level for each competency. Additionally, in their training raters examined PDs’ ratings of video examples to understand their perspective and develop consistency with their thought processes.^11^ On the Electronic Residency Application Service (ERAS®) application, the AAMC provided the numerical score and full video recordings for review. Developers of the SVI sought to provide a more holistic presentation of the applicants beyond traditional test scores.^12^

Given the new data available to PDs, we sought to identify whether a correlation exists between any component of the USMLE examinations and the summative SVI score. Of particular interest was Step 2 CS, which incorporates interpersonal and communication skills into its evaluation. If no correlation between USMLE and SVI scores exists, this suggests that the SVI provides a new piece of information not previously available on the residency application. We hypothesized that numeric Step 1 and Step 2 Clinical Knowledge (CK) scores would not correlate with the SVI score, but that performance on the Step 2 CS portion may correlate with the SVI since both test communication skills.

## METHODS

This was a prospective, cross-sectional study during the 2017–2018 residency application cycle. The study included nine Accreditation Council of Graduate Medical Education (ACGME)-accredited EM residency programs. Each site exported data directly from the ERAS applications, including the SVI score and scores for each component of the USMLE. Unique applicants were identified by their AAMC identification numbers and only included once in the analysis. We included only applicants with an SVI score and at least one score on the USMLE. We studied correlations between USMLE scores and SVI scores. Predetermined subgroup analysis was performed based on applicants’ USMLE Step 1 and Step 2 CK scores as follows: </= 200, 201–220, 221–240, 241–260, >260. USMLE Step 2 CS is graded pass or fail.

We used linear regression to examine correlation between SVI score and USMLE Step 1 and Step 2 CK scores. The Kruskal-Wallis test was used to compare SVI scores with USMLE subcategory scores. We performed the Mann-Whitney U test to compare SVI scores and USMLE Step 2 CS scores.

The study was reviewed by the institutional review board at the primary site.

## RESULTS

A total of 1,329 unique applicants had an SVI score and at least one USMLE step score and were included in the analysis (Table 1). Of these, 1,325 had USMLE Step 1, 1,275 had USMLE Step 2 CK, and 1,000 had USMLE Step 2 CS scores available. Mean scores were as follows: SVI 19.6 (+/− 3.0, range 9–28); USMLE Step 1 231 (+/− 16.0, range 191–273); USMLE Step 2 CK 244 (+/− 14.8, range 188–282).

Using linear regression we found no correlation between the SVI score and overall USMLE Step 1 (p=0.58) or Step 2 CK score (p=0.56, Figure). In subgroup analysis, there was no correlation with specific scores for either Step 1 (p=0.29) or Step 2 CK (p=0.10, Table 2).

Four of the 1,000 students who had a CS score failed the examination. This did not correlate with the SVI score (p=0.08, Table 2).

## DISCUSSION

During the 2017–2018 application season, the SVI score provided an additional objective metric to the EM residency application. This score was intended to measure interpersonal and communication skills, and knowledge of professional behaviors, features not otherwise captured in an objective way on the application. The evaluations of students during their undergraduate medical education are difficult to compare, as schools have varied grading policies and distributions. Data suggest varied correlations between elements of the application and prediction of success in residency, including the USMLE and induction into honor societies such as Alpha Omega Alpha.^13^,^14^

The USMLE provides PDs with a standardized metric as a result of a uniform grading system across all test-takers. In Step 1 and Step 2 CK, examinees answer multiple choice questions related to the basic sciences and then clinical medicine. In this analysis, we found that both overall score on the USMLE as well as individual ranges of score did not correlate with performance on the SVI. Given that the SVI was designed to specifically assess interpersonal and communication skills, as well as knowledge of professional behaviors, it is not surprising that we found no correlation between the USMLE Step 1 and Step 2 CK scores and the SVI score.

The CS portion of the USMLE Step 2, however, assesses communication skills using a standardized patient encounter. Since the SVI also focuses on components of communication, we hypothesized that a correlation could exist between these two scores. In this dataset, only four out of 1,000 (0.4%) applicants who had a CS score available failed the examination. This analysis suggested a trend towards a correlation more than the other analyses; however, it did not achieve statistical significance. Given the extremely low failure rate on the CS examination, it is difficult to assess this correlation. Additionally, the correlation is limited by our inability to break out the analysis by specific USMLE Step 2 CS subcomponent (Communication and Interpersonal Skills; Spoken English Proficiency; and Integrated Clinical Encounter) score ranges. Our results support the notion that the SVI may provide unique information on the residency application. At least in comparison with the USMLE – the only other standard score on the application – we found no correlation between the two scores.

Our results are consistent with those reported by the AAMC.^15^ The AAMC contends that Step 2 CS and the SVI measure related but different constructs.^10^ For example, Step 2 CS measures spoken English proficiency, which is not measured by the SVI. Similarly, the SVI measures teamwork, which is not measured by Step 2 CS. Over time, PDs will benefit from more SVI data including its ability to predict in-person interview scores, success during residency, chief resident selection, and professionalism or communication remediation.

## LIMITATIONS

Given the study design with nine EM residency programs, only 1,329 of the 2,901 total applicants to United States EM residency programs in the 2017–2018 application season were included.^16^ This may limit the overall generalizability of the data set. Additionally, in the cohort of 1,000 applicants in which USMLE Step 2 CS scores were available, only four persons failed the examination, which may have impacted the ability to detect any correlation between this examination and the SVI score.

## CONCLUSION

In this analysis, we found no correlation between the SVI score and any component of the USMLE. As a result, the SVI may provide a unique piece of data for PD interpretation. It is unclear how it will correlate long term with resident performance or success. Additionally, further investigation will help to determine whether the SVI score impacts the decision-making of PDs both in the interview offer and ultimate applicant selection.

## Figures and Tables

**Figure:**
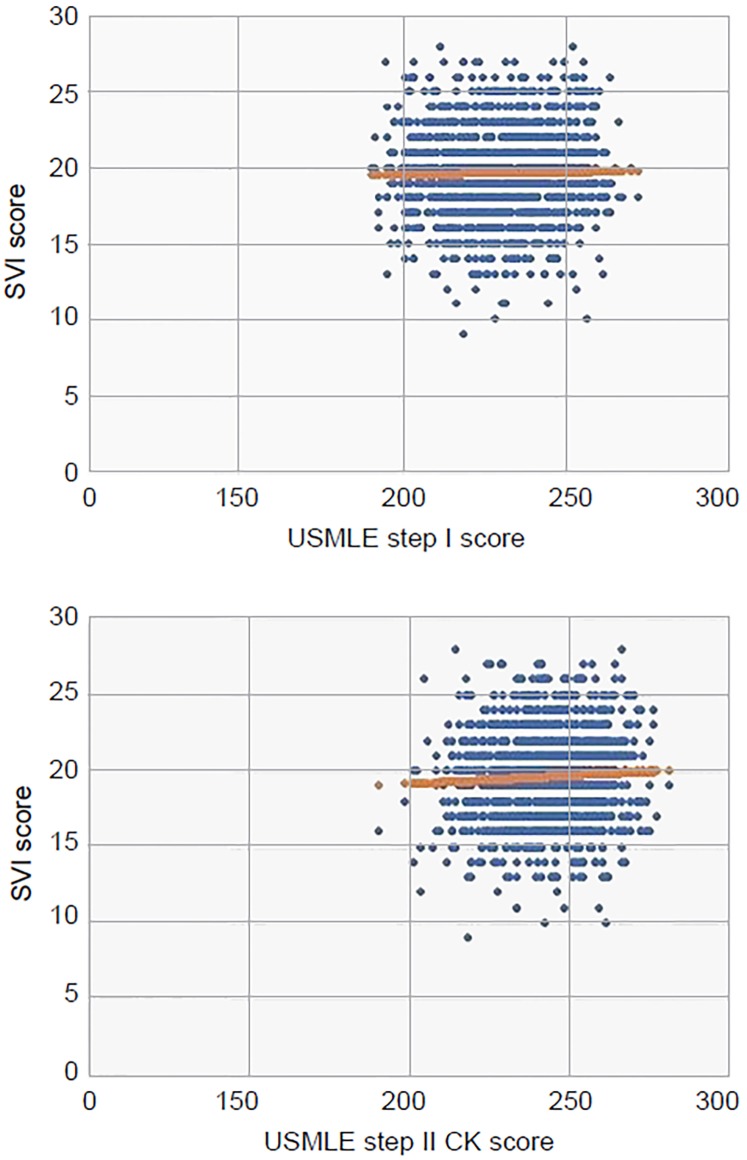
USMLE step I score versus SVI score (top). USMLE step II CK score versus SVI score (bottom). *USMLE*, United States Medical Licensing Examination; *SVI*, standardized video interview; *CK*, clinical knowledge.

**Table 1:** Demographics of emergency medicine residency programs and applicants.

Screener demographics	
Residency programs	
Number of programs	9
University	8 (89%)
Community	1 (11%)
Northeast	4 (40%)
South	3 (30%)
West	2 (30%)
Three-year training programs	7 (78%)
Four-year training programs	2 (22%)
Applicant demographics	n=1329
Mean age	27.6 +/− 3.1 (Range 19–51)
Gender	
Male	64.8%
Medical school location	
Northeast	34.3%
Central	18.7%
South	31.0%
West	12.3%
International	3.3%
Medical school type	
US private	35.7%
US public	51.9%
Osteopathic	9.0%
International	3.3%

*US*, United States.

**Table 2:** USMLE score range with mean SVI score.

	n	Mean SVI (95%CI)	P value
USMLE Step 1 score			
≤200,	30	19.7 (18.6–20.9)	0.29
201–220,	323	19.7 (19.4–20.0)	0.29
221–240,	591	19.5 (19.2–19.7)	0.29
241–260,	354	19.8 (19.5–20.1)	0.29
>260	27	19.0 (18.0–26.0)	0.29
USMLE step 2 CK score			
≤200	3	16.7 (13.8–19.5)	0.10
201–220	98	19.1 (18.4–19.8)	0.10
221–240	402	19.6 (19.3–19.8)	0.10
241–260	610	19.6 (19.4–19.9)	0.10
>260	162	19.8 (19.4–20.3)	0.10
USMLE step 2 CS score			
Pass	996	19.8 (19.6–20.0)	0.08
Fail	4	16.8 (11.5–22.0)	0.08

*USMLE*, United States Medical Licensing Examination; *SVI*, standardized video interview; *CK*, clinical knowledge; *CS*, clinical skills; *CI*, confidence interval.

## References

[1] Hegarty CB, Lane DR, Love JN (2014). Council of Emergency Medicine Residency Directors standardized letter of recommendation writers’ questionnaire. J Grad Med Educ.

[2] Chole RA, Ogden MA (2012). Predictors of future success in otolaryngology residency applicants. Arch Otolaryngol Head Neck Surg.

[3] DeZee KJ, Thomas MR, Mintz M (2009). Letters of recommendation: rating, writing, and reading by clerkship directors of internal medicine. Teach Learn Med.

[4] Shultz K, Mahabir RC, Song J (2012). Evaluation of the current perspectives on letters of recommendation for residency applicants among plastic surgery program directors. Plast Surg Int.

[5] Messner AH, Shimahara E (2008). Letters of recommendation to an otolaryngology/head and neck surgery residency program: their function and the role of gender. Laryngoscope.

[6] Oyama LC, Kwon M, Fernandez JA (2010). Inaccuracy of the global assessment score in the emergency medicine standard letter of recommendation. Acad Emerg Med.

[7] Grall KH, Hiller KM, Stoneking LR (2014). Analysis of the evaluative components on the Standard Letter of Recommendation (SLOR) in Emergency Medicine. West J Emerg Med.

[8] USMLE Step 2 CS.

[9] USMLE FAQs.

[10] AAMC About the SVI.

[11] The AAMC Standardized Video Interview: Essentials for the ERAS®2019 Season.

[12] Bird SB, Blomkalns A, Deiorio NM (2018). Beyond test scores and medical knowledge: the Standardized Video Interview, an Innovative and ethical approach for holistic assessment of applicants. Acad Med.

[13] Bhat R, Takenaka K, Levine B (2015). Predictors of a top performer during emergency medicine residency. J Emerg Med.

[14] Van Meter M, Williams M, Banuelos R (2017). Does the National Resident Match Program rank list predict success in emergency medicine residency programs?. J Emerg Med.

[15] Dunleavy D, Overton R (2018). Standardized Video Interview update.

[16] The MATCH® National Resident Matching Program®. Results and Data 2018 Main Residency Match®.

